# Fecal *Fusobacterium nucleatum* for the diagnosis of colorectal tumor: A systematic review and meta‐analysis

**DOI:** 10.1002/cam4.1850

**Published:** 2019-01-12

**Authors:** Xinyu Zhang, Xiaoqiang Zhu, Yingying Cao, Jing‐Yuan Fang, Jie Hong, Haoyan Chen

**Affiliations:** ^1^ State Key Laboratory for Oncogenes and Related Genes, Division of Gastroenterology and Hepatology, Key Laboratory of Gastroenterology and Hepatology, Ministry of Health, Shanghai Institute of Digestive Disease, Renji Hospital, School of Medicine Shanghai Jiao Tong University Shanghai China

**Keywords:** colorectal cancer, diagnosis, feces, *Fusobacterium nucleatum*

## Abstract

The fecal *Fusobacterium nucleatum* has been reported as a potential noninvasive biomarker for colorectal tumor in several studies, but its exact diagnostic accuracy was ambiguous due to the wide range of sensitivity and specificity. To assess the diagnostic accuracy of fecal *F. nucleatum* for colorectal tumor, we searched electronic databases including PubMed, Cochrane Library, Embase, and Web of Science, without any date and language restrictions. Two reviewers independently extracted data and appraised study quality with Quality Assessment of Diagnostic Accuracy Studies. We included ten studies comprising 13 cohorts for colorectal cancer (CRC) and seven cohorts for colorectal adenoma (CRA). A total of 1450 patients and 1421 controls for CRC and 656 patients and 827 controls for CRA were included. The pooled sensitivity and specificity of fecal *F. nucleatum* for CRC were 71% (95% CI, 61%‐79%) and 76% (95% CI, 66%‐84%), with the area under the receiver‐operating characteristics (AUC) curve of 0.80 (95% CI, 0.76‐0.83). The pooled sensitivity and specificity of fecal *F. nucleatum* for CRA were 36% (95% CI, 27%‐46%) and 73% (95% CI, 65%‐79%), with an AUC of 0.60 (95% CI, 0.56‐0.65). Substantial heterogeneity among studies existed, which was partly caused by DNA extraction kits, regions of study, sample size, and demographic characteristics of participants. Fecal *F. nucleatum* was valuable for the diagnosis of CRC although it performed below expectation. For CRA, the specificity of fecal *F. nucleatum* indicated the possibility of noninvasive screening. Subgroup analyses for adenoma were incomplete due to lack of data. Heterogeneity limited the credibility of the study.

## 1 INTRODUCTION

Colorectal cancer (CRC) is the third most common cancer in the world and ranks as one of the five most common fatal cancers worldwide.^1^ The high morbidity and mortality are mainly due to the fact that CRC is usually not diagnosed until it has reached an advanced stage. Both incidence and death rates of CRC declined during recent years, largely thanks to the use of screening methods.^2^ Several screening strategies including colonoscopy and fecal immunochemical test (FIT) are recommended by international guidelines.^3^, ^4^ However, due to economic limitations and screening process, a majority of people has not undergone colonoscopy.^3^ On the other hand, FIT had a large range of the sensitivity for CRC, from 25% to 100%,^5^ and worked weakly when detecting colorectal adenoma (CRA),^6^, ^7^, ^8^, ^9^ although it has been a widely accepted screening strategy for CRC. Moreover, some conditions such as hemorrhoids could increase the risk of false‐positive FIT results.^10^ Thus, more noninvasive and economic biomarkers for CRC detection are urgently needed.

Recently, increased attention has been paid to the effect of microbiome in CRC. Since *Fusobacterium nucleatum* (*F. nucleatum*) infection was reported to be prevalent in CRC tissues,^11^ many studies have focused on its role in the carcinogenesis and development of colorectal tumor.^12^ In addition, *F. nucleatum* was also detected significantly more in the feces of CRC patients than healthy controls,^13^ suggesting that fecal *F. nucleatum* may be helpful for noninvasive CRC screening. Fecal *F. nucleatum* has been reported as a potential novel biomarker for CRC, even with a higher detection rate for CRC than FIT.^14^, ^15^ Another study also reported that the diagnostic accuracy of fecal *F. nucleatum* for CRC was as well as that of FIT, with a better diagnostic accuracy for CRA.^16^ These studies were encouraging, indicating that fecal *F. nucleatum* would be a promising biomarker for colorectal tumor and even be comparable with FIT. However, there were also some studies producing conflicting results.^17^, ^18^, ^19^ The diagnostic characteristics of fecal *F. nucleatum* for CRC have been ambiguous, with sensitivity ranging from 45% to 100% and specificity ranging from 10% to 92%.^15^, ^17^, ^18^ These all have added difficulties to assess the diagnostic accuracy of fecal *F. nucleatum* for colorectal tumor. Therefore, we conducted a meta‐analysis to explore the diagnostic accuracy of fecal *F. nucleatum* for CRC or CRA.

## 2 METHODS

### 2.1 Search strategy

We did a systematic search of several electronic databases, including PubMed, Cochrane Library, Embase, and Web of Science, without any date and language restrictions, for all studies about diagnostic performance of fecal *F. nucleatum* for CRC or CRA. We used medical subject headings and keywords for literature retrieval. The last search was performed in June 2018. Further details of the search strategy are provided in Supplementary Method.

### 2.2 Selection criteria

Two reviewers independently checked titles and abstracts of all retrieved articles and determined final eligibility according to full texts. All disagreement was settled by discussion and reached consensus. Studies were included if they met the following criteria: (a) studies evaluated the diagnostic accuracy of fecal *F. nucleatum* for CRC or CRA; (b) studies presented sufficient data to infer a two‐by‐two diagnostic table, comprising true positives (tp), false positives (fp), false negatives (fn), and true negatives (tn). We excluded letters, reviews, conference abstracts, and duplicate publications.

### 2.3 Data extraction and quality assessment

The same reviewers independently extracted study data to obtain relevant information. We contacted study authors to achieve data for extraction when necessary. For studies presenting results with different cutoff values,^16^ different subspecies^18^ and different subject‐recruiting sites,^15^, ^19^ we extracted all data to get the most information of these studies. The revised Quality Assessment of Diagnostic Accuracy Studies (QADAS‐2)^20^ was used to score the quality of included studies, and discrepancies were resolved.

### 2.4 Data synthesis and analysis

We performed data synthesis and analysis with STATA 14.0 (StataCorp, College Station, Texas), using midas and metandi modules. *P* value was regarded as statistically significant when it was equal to or less than 0.05. We calculated the pooled sensitivity, specificity, positive‐likelihood ratio, negative‐likelihood ratio, diagnostic odds ratio (DOR), and summary receiver‐operating characteristics (SROC) curve with 95% confidence interval (CI) by using hierarchical models. Hierarchical models include the bivariate model^21^ and the hierarchical SROC model.^22^ The former directly models the sensitivity, specificity and the correlation between them. The latter defines a hierarchical SROC (HSROC) curve by modeling functions of sensitivity and specificity.

We used a Deeks’ funnel plot to test the publication bias. It tests association between log diagnostic odds ratio (lnDOR) and “effective sample size,” which is a sample function of diseased and nondiseased individuals.^23^ The slope coefficient and relevant *P* value are associated with publication bias. We explored heterogeneity among included studies because study characteristics may be related to the study size and test accuracy.^23^ The I‐square (*I* ^2^) was calculated to estimate the heterogeneity.^24^

In our analysis, we used the one with higher sensitivity for the study^18^ presenting results with two different subspecies; we used the combined cohort, which included discovery cohort and validation cohort, for the study^16^ presenting results with three cohorts (discovery cohort, validation cohort, and combined cohort) and different cutoff values.

We excluded study one by one to assess the robustness of our findings.

We performed a series of predefined subgroup analyses according to DNA extraction kits (QIGEN or not QIGEN), regions (Asia or non‐Asia), and sample size (<200 or >200). Cutoff values and internal controls were different among included studies, so we could not stratify studies by them. Furthermore, we conducted a bivariate random‐effects meta‐regression analysis to explore the sources of heterogeneity further, with the following variables: the percent of early‐stage patients in all CRC patients (<50% or >50%), the percent of patients in all participants (<50% or 50%), the percent of males in patients (<60% or >60%), and the average age of all participants.

## 3 RESULTS

### 3.1 Study selection

**Figure 1:**
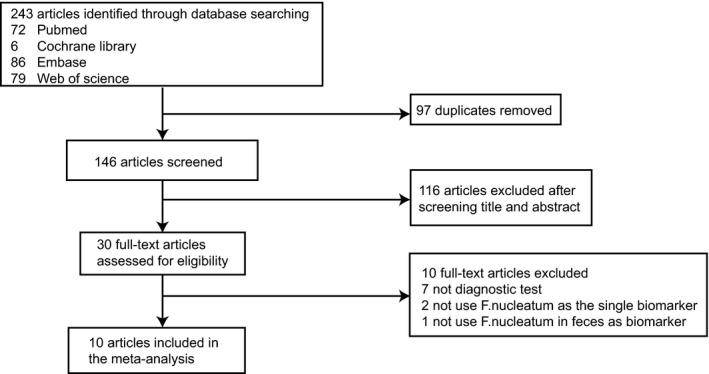
Flow diagram of study selection

We retrieved 243 articles through electronic databases at first, comprising 72 articles from PubMed, six articles from Cochrane Library, 86 articles from Embase, and 79 articles from Web of Science. After removing duplicates, we screened titles and abstracts of 146 articles and excluded 116 articles. We read full text of 30 studies, and 10 studies^13^, ^14^, ^15^, ^16^, ^17^, ^18^, ^19^, ^25^, ^26^, ^27^ were eligible in the meta‐analysis in the end and six^14^, ^16^, ^17^, ^19^, ^25^, ^26^ of them also reported diagnostic results of CRA. We included 13 cohorts of CRC and seven cohorts of CRA in the end because three articles^15^, ^19^, ^27^ recruited two cohorts independently from different sites. The procedure of study selection is shown in Figure 1.

### 3.2 Study characteristics

The main characteristics of 13 cohorts of CRC and seven cohorts of CRA are shown in Table 1.

**Table 1:** Main characteristics of included studies in the meta‐analysis

Study, year	Region	Participants, no.	Patients, no.	Standard reference	Mean age (control)	Mean age (patients)	Percent of early‐stage patients^a^, %	Percent of males^b^, %	DNA extraction kit
CRC									
Zellar et al, 2014^24^	France	141	53	Endoscopy	60.49	66.81	41.51	53.19	GNOME DNA Isolation Kit
Mira‐Pascual et al, 2015^32^	Spain	16	7	Colonoscopy	52.6	71.14	71.43	76.47	Commercial kits
Fukugaiti et al, 2015^19^	Brazil	17	7	Colonoscopy	54.8	65.4	NA	76.47	QiAamp DNA Stool Mini Kit
Suehiro et al, 2016^31^	Japan	218	158	NA^c^	32	69	49.37‐52.53^d^	NA^c^	QiAamp DNA Stool Mini Kit
Amitay et al, 2017^23^	Germany	237	44	Endoscopy	NA^e^	NA^e^	50.00‐54.55^e^	42.62‐59.49^e^	FastDNA Spin Kit for Soil
Eklof et al, 2017^20^	Sweden	104	39	Patient records^f^	>60^g^	>60^g^	60.53	52.88	QiAamp DNA Stool Mini Kit
Liang et al, 2017^21^	Hongkong, China	370	170	Colonoscopy and histopathology	59.3	67.2	55.49	47.84	QiAamp DNA Stool Mini Kit
Liang et al, 2017^21^	China	69	33	Colonoscopy and histopathology	53.2	63.4	42.42	39.13	QiAamp DNA Stool Mini Kit
Wong et al, 2017^22^	Hongkong, China	325	127	Endoscopy and pathology	57.83	66.34	NA^c^	61.85	ZR Faecal DNA MiniPrep Kit
Xie et al, 2017^25^	China	569	327	Endoscopy and pathology	59.49	63.13	33.33	57.64	QiAamp DNA Stool Mini Kit
Xie et al, 2017^25^	China	180	118	Endoscopy and pathology	62.58	64.66	29.97	61.67	QiAamp DNA Stool Mini Kit
Guo et al, 2018^33^	China	371	215	Histopathology	48.6	61.2	NA	50.67	DNA Stool Mini Kit
Guo et al, 2018^33^	China	254	152	Histopathology	51.7	56.4	NA	58.66	DNA Stool Mini Kit
CRA									
Mira‐Pascual et al, 2015^32^	Spain	17	8	Colonoscopy	52.6	63.27	NA	76.20	Commercial kits
Suehiro et al, 2016^31^	Japan	90	30	NA^c^	32	67.53	36.67	NA^c^	QiAamp DNA Stool Mini Kit
Amitay et al, 2017^23^	Germany	386	193	Endoscopy	NA^e^	NA^e^	51.30	56.48‐72.02^e^	FastDNA Spin Kit for Soil
Eklof et al, 2017^20^	Sweden	199	134	Patient records^e^	>60^g^	>60^h^	92.59	57.79	QiAamp DNA Stool Mini Kit
Wong et al, 2017^22^	Hongkong, China	363	165	Endoscopy and pathology	57.83	60.10	0	63.64	ZR Faecal DNA MiniPrep Kit
Xie et al, 2017^25^	China	344	102	Endoscopy and pathology	59.49	NA^c^	NA^c^	NA^c^	QiAamp DNA Stool Mini Kit
Xie et al, 2017^25^	China	84	24	Endoscopy and pathology	62.58	NA^c^	NA^c^	NA^c^	QiAamp DNA Stool Mini Kit

CRC, colorectal cancer; CRA, colorectal adenoma; NA, not available.

a Percent of advanced CRC patients in all CRC patients.

b Percent of males in all participants.

c Data are not available.

d The stage of five patients is not available, so we calculated the largest and smallest possible percentage of advanced patients.

e Participants included in PCR analysis were less than overall study participants and in the article of Amitay et al, only overall study participants’ characteristics were given. So the mean age was not available and we calculated the largest and smallest possible percentage of advanced patients and males.

f Included pathology records.

g Mean age >60 y because only 10% of the control and CRC patients were aged 34‐59 y.

h Mean age >60 y because only 17.2% percent of colorectal neoplasia patients were aged 34‐59 y.

Other than one^25^ of these studies did not mention the exclusive criteria; the other studies^13^, ^14^, ^15^, ^16^, ^17^, ^18^, ^19^, ^26^, ^27^ all excluded patients with conditions (such as a vegetarian diet or use of antibiotics within the recent 3 months) that may influence the intestinal microbiome or medical conditions (such as inflammatory bowel disease or a history of cancer) which were relevant to colorectal tumor. One^18^ of these studies grouped patients with small adenoma into the control. All of these studies were case‐control study. One^25^ study counted the absolute copy number of fecal *F. nucleatum*, while the other studies^13^, ^14^, ^15^, ^16^, ^17^, ^18^, ^19^, ^26^, ^27^ detected the relative abundance of it. However, studies evaluating the relative abundance of fecal *F. nucleatum* chose different internal controls.

We analyzed data for CRC firstly. These studies included 1450 patients and 1421 controls. Sample sizes of cohorts ranged from 16 to 569. The exact cutoff values were available in four studies^14^, ^15^, ^16^, ^25^ and different from each other.

The data available for CRA were fewer. In total, 652 patients with adenoma and 827 controls were included in the evaluation of diagnostic ability of fecal *F. nucleatum* for CRA. Sample sizes of cohorts varied widely, ranging from 17 to 386. The exact cutoff values were reported in three studies^14^, ^16^, ^25^ and also different. Three^14^, ^17^, ^25^ studies used the same cutoff values to diagnose adenoma as to diagnose CRC.

### 3.3 Quality assessment

The results of the QADAS‐2 for CRC and CRA are shown in Figure S1 and Table S1, indicating that highest risk of bias existed in “patient selection” and “index text.” The former one is because that all of the included studies were case‐control study and the percentage of colorectal tumor patients in all subjects was inconsistent with its prevalence rate. However, except for the one^25^ that did not mention the way of subject selection, the other studies all recruited participants consecutively or randomly. The latter one is caused by cutoff values not being determined beforehand in all studies but one.^18^ The highest concern about applicability came from “patient selection.” Four studies^14^, ^15^, ^16^, ^18^ included subjects with gastrointestinal symptoms such as changes of bowel movement or visible bleeding. Four studies^15^, ^16^, ^17^, ^26^ included participants aged 50 years or older. Two studies^13^, ^19^ included participants aged 40 years or above. One study included asympotomatic participants.^26^ One study^25^ did not report specific information about the inclusion and exclusion criteria of study cohort.

### 3.4 Assessment of publication bias and heterogeneity

The publication bias of studies for CRC and CRA is displayed in Figure S2. Both of the two funnel plots were almost symmetrical. *P* values of slope coefficient of the two regression lines were 0.7 and 0.67, both more than 0.1, suggesting a low likelihood of publication bias. Obvious heterogeneity existed when we calculated pooled sensitivity (*I* ^2^ = 88.45%), specificity (*I* ^2^ = 86.44%), positive DLR (*I* ^2^ = 83.57%), and negative DLR (*I* ^2^ = 85.83%) to evaluate diagnostic ability of fecal *F. nucleatum* for CRC. The proportion of heterogeneity likely due to threshold effect was 31%. When it comes to CRA, substantial heterogeneity also existed in pooled sensitivity (*I* ^2^ = 86.18%), specificity (*I* ^2^ = 65.79%), positive DLR (*I* ^2^ = 47.61%), and negative DLR (*I* ^2^ = 72.96%). The percent of heterogeneity owing to threshold effect was 13%. Different thresholds among studies contributed to the heterogeneity limitedly for both CRC and CRA.

### 3.5 Overall diagnostic accuracy

**Figure 2:**
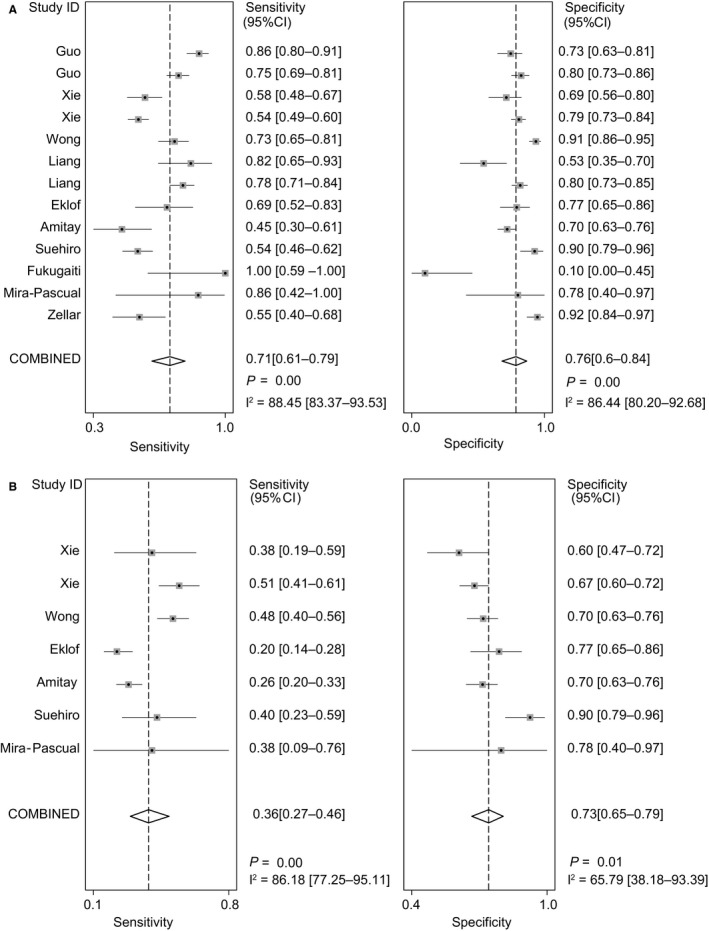
Pooled sensitivity and specificity for colorectal cancer and colorectal adenoma. Forest plots demonstrate the pooled sensitivity and specificity of fecal *Fusobacterium nucleatum* for A, colorectal cancer and B, colorectal adenoma. CI, confidence interval

**Figure 3:**
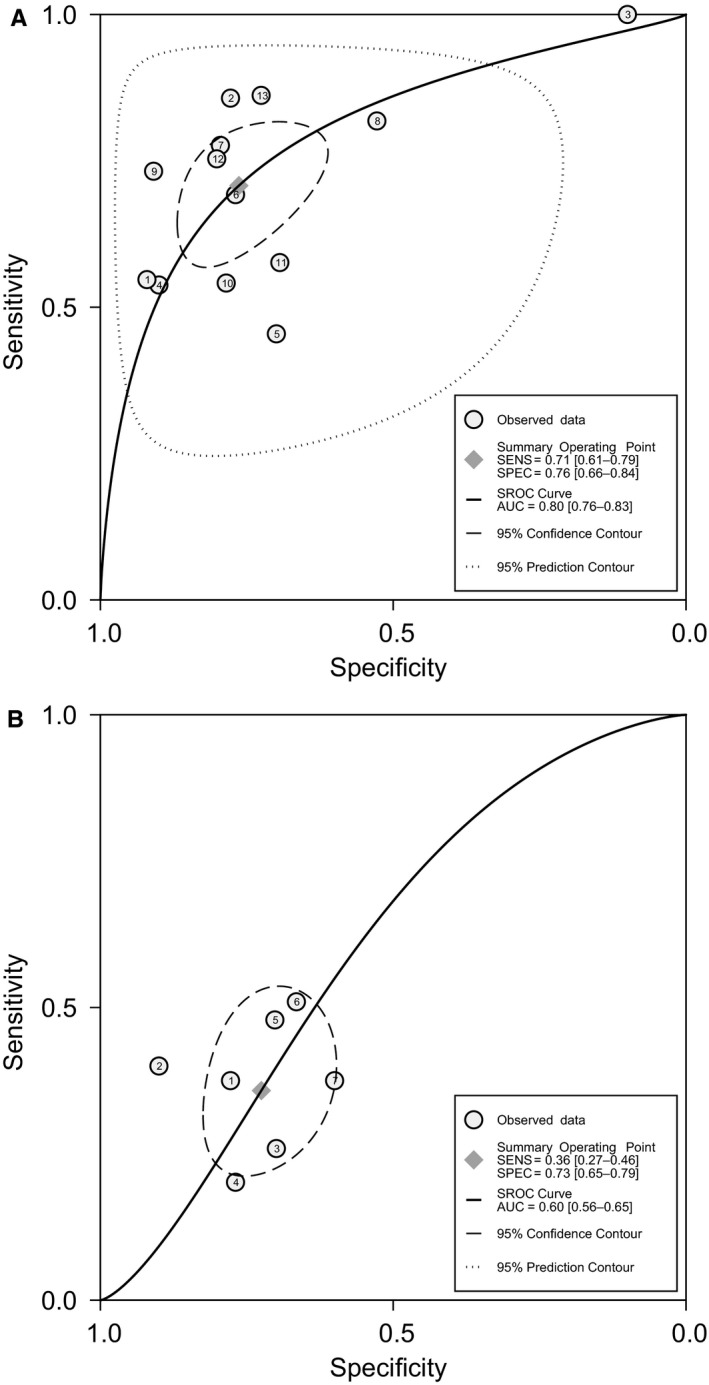
HSROC curve of sensitivity versus specificity of fecal *Fusobacterium nucleatum* for colorectal cancer and adenoma. HSROC curve of fecal *Fusobacterium nucleatum* for diagnosis of A, colorectal cancer and B, colorectal adenoma. HSROC curve, hierarchical receiver‐operating characteristics curve

The forest plot displayed sensitivity of 71% (95% CI, 61%‐79%) and specificity of 76% (95% CI, 66%‐84%) for CRC (Figure 2). The pooled sensitivity for CRA was 36% (95% CI, 27%‐46%), much lower than that for CRC (Figure 2). And the specificity for CRA was 73% (95% CI, 65%‐79%) (Figure 2). The SROC curve yielded a moderate area under the curve (AUC) of 0.80 (95% CI, 0.76‐0.83) for CRC and a low AUC of 0.60 (95% CI, 0.56‐0.65) for CRA (Figure 3). The pooled estimates of positive DLR and negative DLR for CRC were 2.99 (95% CI, 2.13‐4.20) and 0.38 (95% CI, 0.29‐0.50), respectively (Figure S3). The same parameters for CRA were 1.3 (95% CI, 0.95‐1.78) and 0.89 (95% CI, 0.76‐1.03) (Figure S4), consistent with low sensitivity and AUC of fecal *F. nucleatum* for CRA.

### 3.6 Sensitivity analysis

We explored the robustness of our results by removing studies one by one. The results of sensitivity analyses are shown in Table 2.

**Table 2:** Sensitivity analyses for the robustness of pooled results

Study, year	Participants, No.	Sensitivity, % (95% CI)	Specificity, % (95% CI)	Positive DLR (95% CI)	Negative DLR (95% CI)	AUC (95% CI)
CRC						
Zellar et al, 2014^24^	2730	72 (62‐80)	75 (64‐83)	2.8 (2.0‐3.9)	0.38 (0.28‐0.50)	0.79 (0.76‐0.83)
Mira‐Pascual et al, 2015^32^	2855	70 (60‐78)	76 (65‐85)	3.0 (2.1‐4.2)	0.39 (0.30‐0.51)	0.79 (0.75‐0.83)
Fukugaiti et al, 2015^19^	2854	68 (60‐76)	79 (73‐85)	3.3 (2.5‐4.4)	0.40 (0.31‐0.51)	0.81 (0.77‐0.84)
Suehiro et al, 2016^31^	2653	NA	NA	NA	NA	NA
Amitay et al, 2017^23^	2634	81 (77‐84)	77 (66‐85)	3.2 (2.2‐4.5)	0.35 (0.27‐0.46)	0.81 (0.77‐0.84)
Eklof et al, 2017^20^	2767	71 (61‐80)	76 (65‐85)	3.0 (2.1‐4.3)	0.38 (0.28‐0.51)	0.80 (0.76‐0.83)
Liang et al, 2017^21^	2501	70 (60‐79)	76 (64‐85)	2.9 (2.0‐4.2)	0.39 (0.30‐0.52)	0.79 (0.75‐0.82)
Liang et al, 2017^21^	2802	70 (60‐78)	78 (68‐85)	3.2 (2.2‐4.5)	0.39 (0.30‐0.51)	0.80 (0.76‐0.83)
Wong et al, 2017^22^	2546	71 (60‐80)	74 (64‐83)	2.8 (2.0‐3.8)	0.39 (0.29‐0.52)	0.79 (0.75‐0.82)
Xie et al, 2017^25^	2302	72 (63‐80)	76 (65‐85)	3.0 (2.1‐4.4)	0.36 (0.27‐0.48)	0.80 (0.77‐0.84)
Xie et al, 2017^25^	2691	72 (62‐81)	77 (66‐85)	3.1 (2.2‐4.5)	0.36 (0.27‐0.48)	0.81 (0.77‐0.84)
Guo et al, 2018^33^	2500	79 (75‐82)	76 (64‐85)	2.9 (2.0‐4.2)	0.39 (0.29‐0.52)	0.79 (0.75‐0.82)
Guo et al, 2018^33^	2617	68 (59‐76)	77 (65‐85)	2.9 (2.0‐4.3)	0.41 (0.33‐0.53)	0.77 (0.74‐0.81)
CRA						
Mira‐Pascual et al, 2015^32^	1466	36 (26‐47)	72 (65‐79)	1.3 (0.9‐1.8)	0.89 (0.75‐1.04)	0.60 (0.56‐0.64)
Suehiro et al, 2016^31^	1393	36 (26‐47)	69 (65‐73)	1.2 (9‐1.5)	0.93 (0.79‐1.08)	0.67 (0.63‐0.71)
Amitay et al, 2017^23^	1097	38 (28‐49)	38 (28‐49)	1.5 (1.0‐2.0)	0.84 (0.71‐0.99)	0.61 (0.56‐0.65)
Eklof et al, 2017^20^	1284	40 (31‐49)	72 (63‐79)	1.4 (1.0‐2.0)	0.84 (0.70‐1.00)	0.58 (0.54‐0.62)
Wong et al, 2017^22^	1120	33 (24‐44)	73 (64‐81)	1.3 (0.8‐1.9)	0.91 (0.76‐1.08)	0.56 (0.52‐0.61)
Xie et al, 2017^25^	1139	33 (24‐43)	74 (65‐81)	1.3 (0.8‐1.9)	0.90 (0.76‐1.08)	0.56 (0.52‐0.61)
Xie et al, 2017^25^	1399	35 (25‐47)	74 (67‐0.80)	1.4 (1.0‐1.9)	0.87 (0.74‐1.03)	0.63 (0.59‐0.67)

CRC, colorectal cancer; CRA, colorectal adenoma; NA, not available.

### 3.7 Subgroup analysis

Table 3 summarizes all results of the subgroup analyses for diagnosis of CRC and CRA.

**Table 3:** Results of subgroup analyses depending on DNA extraction kit, region, and sample size

Characteristic	Cohorts, No.	Participants, No.	Sensitivity, % (95% CI)	*I* ^2^, %	Specificity, % (95% CI)	*I* ^2^, %	Positive DLR (95% CI)	*I* ^2^, %	Negative DLR (95% CI)	*I* ^2^, %	AUC
CRC											
DNA extraction kit											
QIAGEN	7	1527	72 (56‐84)	85.70	69 (50‐84)	86.46	2.3 (1.5‐3.6)	79.92	0.41 (0.28‐0.59)	74.82	0.77 (0.73‐0.80)
Not QIAGEN	6	1344	71 (58‐81)	87.60	82 (73‐89)	88.34	4.0 (2.5‐6.4)	77.67	0.35 (0.23‐0.53)	89.56	0.84 (0.81‐0.87)
Region											
Asia	8	2356	71 (62‐79)	92.22	79 (71‐85)	83.63	3.4 (2.4‐4.7)	71.99	0.37 (0.27‐0.49)	90.45	0.82 (0.78‐0.85)
Non‐Asia	5	515	71 (44‐88)	67.62	68 (37‐89)	90.51	2.2 (1.1‐4.6)	85.45	0.43 (0.23‐0.81)	57.86	0.75 (0.71‐0.79)
Sample size											
>200	7	2344	68 (57‐78)	57.29	81 (75‐86)	86.69	3.6 (2.6‐5.0)	59.32	0.45 (0.31‐0.66)	42.51	0.83 (0.79‐0.86)
<200	6	527	72 (55‐85)	65.92	66 (40‐85)	89.00	2.1 (1.2‐3.7)	80.58	0.42 (0.28‐0.63)	8.64	0.75 (0.71‐0.79)
Colorectal adenoma											
DNA extraction kit											
QIAGEN	4	717	36 (24‐50)	87.93	75 (61‐84)	82.57	1.40 (0.85‐2.31)	48.54	0.86 (0.69‐1.08)	72.10	0.57 (0.53‐0.61)
Not QIAGEN	3	766	NA	NA	NA	NA	NA	NA	NA	NA	NA
Region											
Asia	4	881	47 (41‐53)	0.00	73 (60‐83)	80.80	1.72 (1.16‐2.54)	13.38	0.73 (0.63‐0.85)	16.58	0.52 (0.47‐0.56)
Not Asia	3	602	NA	NA	NA	NA	NA	NA	NA	NA	NA
Sample size											
>100	4	1292	35 (23‐50)	93.10	70 (66‐74)	0	1.2 (0.8‐1.7)	63.22	0.93 (0.77‐1.12)	82.59	0.67 (0.63‐0.71)
<100	3	191	NA	NA	NA	NA	NA	NA	NA	NA	NA

CRC, colorectal cancer; DLR, diagnostic likelihood ratio; Fn, *Fusobacterium nucleatum*; NA, Insufficient data for pooling results.

### 3.8 DNA extraction kit

The pooled sensitivity of studies^13^, ^14^, ^15^, ^19^, ^25^ using QiAamp DNA Stool Mini Kit (QIAGEN group) to diagnose CRC was 72%, which was similar to that of studies not using QiAamp DNA Stool Mini Kit (not QIAGEN group), with a lower specificity and positive DLR compared with those of not QIAGEN group. The AUC of the QIAGEN group was lower than that of the not QIAGEN group. The heterogeneity of positive DLR and negative DLR decreased, while the heterogeneity of sensitivity and specificity kept unchanged.

For CRA, studies^14^, ^19^, ^25^ using QiAamp DNA Stool Mini Kit had a slightly higher specificity and positive DLR and lower negative DLR, with unchanged sensitivity, compared with overall estimates. However, the heterogeneity increased in the subgroup analyses. We did not calculate the pooled diagnostic accuracy of the other three studies^16^, ^17^, ^26^ not using QiAamp DNA Stool Mini Kit because of limited data.

### 3.9 Region

We calculated the pooled diagnostic accuracy of fecal *F. nucleatum* for Asian studies.^15^, ^16^, ^19^, ^25^, ^27^ For CRC, the pooled diagnostic accuracy of Asian studies was better than that of non‐Asian studies. But the heterogeneity of sensitivity and negative DLR in non‐Asian studies decreased significantly.

For CRA, compared with the overall pooled results, the pooled sensitivity and positive DLR of Asian^16^, ^19^, ^25^ studies were higher, especially the sensitivity, with the lower pooled negative DLR. Furthermore, heterogeneity of these indicators all decreased significantly except that of specificity.

### 3.10 Sample size

For cohorts with larger sample size^15^, ^16^, ^17^, ^19^, ^25^, ^27^ (>200), the diagnostic ability of fecal *F. nucleatum* for CRC was better than cohorts^13^, ^14^, ^15^, ^18^, ^19^, ^26^ with smaller sample size, with lower sensitivity. And the heterogeneity of sensitivity, positive DLR, and negative DLR all dropped sharply. For CRA, heterogeneity of specificity did not exist among cohorts with larger sample size^14^, ^16^, ^17^, ^19^ (>100) and their pooled AUC was better than the overall one.

### 3.11 Meta‐regression

Studies of CRA diagnosis had limited information, so we only conducted a meta‐regression for CRC. The meta‐regression showed that the average age of all participants contributed to the heterogeneity of sensitivity. Besides, the percent of early‐stage patients in all CRC patients, the percent of the males in all participants, and the average age of all participants were responsible for the overall heterogeneity (Table S2).

## 4 DISCUSSION

There have been many studies investigating the mechanism of *F. nucleatum* instigating and potentiating colorectal tumor by using tumor tissues. It has been reported that the accumulation of *F. nucleatum* in colorectal tumor is partly due to fusobacterial lectin Fap2^28^ and FadA adhesin^29^ and that *F. nucleatum* can induce the carcinogenesis and development of colorectal tumor by the microRNA‐21‐mediated pathway^30^ or inhibition of host adaptive immunity,^31^, ^32^, ^33^ etc Recently, our group also found that *F. nucleatum* promoted chemoresistance by modulating autophagy in CRC.^34^ *F. nucleatum* was reported to be associated with the prognosis of CRC as well.^35^, ^36^ There also have been some studies exploring diagnosing colorectal tumor with *F. nucleatum* from tumor tissues^26^, ^37^ or feces. However, it was reported that the level of *F. nucleatum* in feces did not correlate with that in tumor tissues.^38^ Our study focused on the fecal *F. nucleatum* to explore its potential as a noninvasive screening method for colorectal tumor.

In our meta‐analysis, the overall pooled diagnostic accuracy of fecal *F. nucleatum* (AUC) was 0.80 for CRC; the pooled sensitivity and specificity of fecal *F. nucleatum* was 71% and 76% for CRC, indicating that fecal *F. nucleatum* has a certain value for the diagnosis of CRC. The pooled sensitivity (36%), specificity (73%), and AUC (0.60) for CRA were low. It is worth noting that the diagnostic performance of FIT for CRA was also poor, with sensitivity ranging from 15% to 26.3%.^7^, ^9^ What is more, it cannot be denied that FIT has many limitations, such as false‐positive results mentioned above. And FIT could not diagnose patients with nonbleeding lesions,^14^ which could be complemented by fecal *F. nucleatum*. It was reported that combining fecal *F. nucleatum* with FIT or other microbial markers enhanced the detection ability of FIT for both CRC and CRA.^15^, ^16^, ^39^ Thus, it is hopeful for *F. nucleatum* to be a biomarker for the noninvasive screening of colorectal tumor.

We conducted sensitivity analyses excluding studies one by one and found our results were stable, especially for CRC.

To investigate the source of heterogeneity, we did subgroup analyses and meta‐regression. The DNA extraction kits, cohort regions, sample size, average age, and the percent of early‐stage patients and the males were responsible for the high heterogeneity. The percent of heterogeneity owing to threshold effect was low.

It was reported that variations in DNA extraction protocol had a great impact on the observation of fecal microbial composition.^40^ It is consistent with the better diagnostic accuracy of not QIAGEN group for CRC compared with QIAGEN group. However, high heterogeneity among studies using the same DNA extraction kit still existed although it decreased comparing with the overall one. For CRA, QIAGEN group also existed high heterogeneity of sensitivity and specificity despite decreased heterogeneity of DLR. Besides the difference of DNA extraction kit, we found that methods of storing fecal samples before DNA extracting, targeted genes, primers’ sequences designed for PCR, and internal controls were different among studies as well. But we could not analyze their exact effect on heterogeneity due to limited data. Further studies are needed to explore whether these factors are responsible for heterogeneity and find the best way to utilize fecal *F. nucleatum* for diagnosis of colorectal tumor.

The diagnostic accuracy of fecal *F. nucleatum* for CRC in Asian studies was better compared with diagnostic accuracy in non‐Asian studies. And the heterogeneity also decreased to some extent. For CRA, the heterogeneity of sensitivity even dropped to zero. There was evidence suggesting that intestinal *F. nucleatum* could be influenced by diet.^41^ Other studies also reported that the change of diet could alter intestinal microbiome.^42^, ^43^ That may be associated with the different diagnostic accuracy among different regions and the decreased heterogeneity in region subgroups.

In addition, the subgroup analysis according to sample size indicated that the fecal *F. nucleatum* had performed better for the diagnosis of colorectal tumor in larger cohorts and sample size was an important factor for heterogeneity.

The percent of early‐stage patients and males and the average age were the source of heterogeneity as well. It was reported that copy number of fecal *F. nucleatum* was the highest in stage II and the lowest in stage IV.^25^ Another study also reported that the relative abundance of fecal *F. nucleatum* in CRC stages II and III was higher than stage I.^39^ This may explain the effect of stage composition on heterogeneity. Enrichment of fecal *F. nucleatum* in CRC stage II suggested that fecal *F. nucleatum* may play a role in detecting early‐stage CRC.

Furthermore, it is a remarkable fact that colorectal tumor is a disease due to different genetic and epigenetic alterations, such as microsatellite instability and activation of oncogenes including KRAS and BRAF. It was also reported that *F. nucleatum*, which was influenced by food, lifestyle, and medications, was related with clinical and molecular pathologies of colorectal tumor.^16^, ^36^ Molecular pathological epidemiology (MPE), a transdisciplinary and interdisciplinary integrative field, studies associations of an exposure with molecular or pathological features of a certain disease.^44^ In view of this, microbial MPE also contributes to the heterogeneity of colorectal tumor as well as our study. In addition, in the era of precision medicine, MPE has the potential to play an important role in the future.^45^ Thus, combining MPE research with fecal bacterial analyses, such as *F. nucleatum*, may advance investigations of pathogenesis of colorectal tumor, help diagnose or classify this disease, and improve the development of precision medicine for colorectal tumor.

Our study has some limitations. First, the sizes of studies and samples for CRA were small, despite the use of a comprehensive search strategy. This limited the precision of pooled results and prevented complete subgroup analyses for CRA.

All of these studies were case‐control designs, causing spectrum effects by restricting sampling of cases and controls.^46^ That were likely to lead to high bias and inflated estimates of results. And some included studies had small sample size and did not conform to the principles of diagnostic tests such as blind control.^47^ All above did harm to the validity of results in these studies.

What is more, there were no unified detecting methods and threshold because assessing the diagnostic accuracy of fecal *F. nucleatum* just emerged over the past few years. The sensitivity and specificity were determined by the threshold of AUC curve. To some extent, the sensitivity and specificity are mutually dependent, where lowering the threshold to increase sensitivity will decrease specificity and vice versa. Most of the included studies defined their own threshold by their ROC curve and obtained different cutoff values. In terms of this, sensitivity or specificity in these studies was not parallel. For these reasons, further studies with rigorous design and randomized clinical trials are needed to define the best cutoff value for the diagnosis of colorectal tumor with fecal *F. nucleatum* and assess its performance.

In conclusion, fecal *F. nucleatum* is promising for the noninvasive diagnosis of colorectal tumor. It is a potential complementary method of FIT, especially for CRA. Given the results of subgroup analyses and meta‐regression, further studies should be performed to determine standard *F. nucleatum* detecting methods and diagnostic threshold to reduce heterogeneity and enhance clinical effectiveness of fecal *F. nucleatum*.

## CONFLICT OF INTEREST

None declared.
