# Supplementary figures and images for: Fecal Fusobacterium nucleatum for the diagnosis of colorectal tumor: A systematic review and meta‐analysis
Source: Cancer Med. 2019 Jan 12;8(2):480–91. doi: 10.1002/cam4.1850 (PMC6382715; doi:10.1002/cam4.1850)

Figure S1: Overall quality assessment of included studies according to QADAS-2 criteria

A

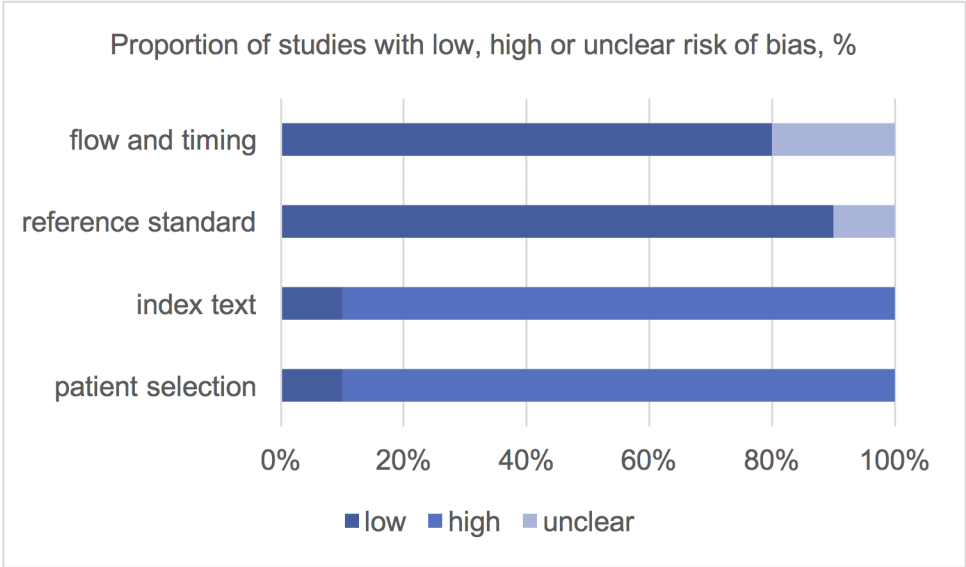

B

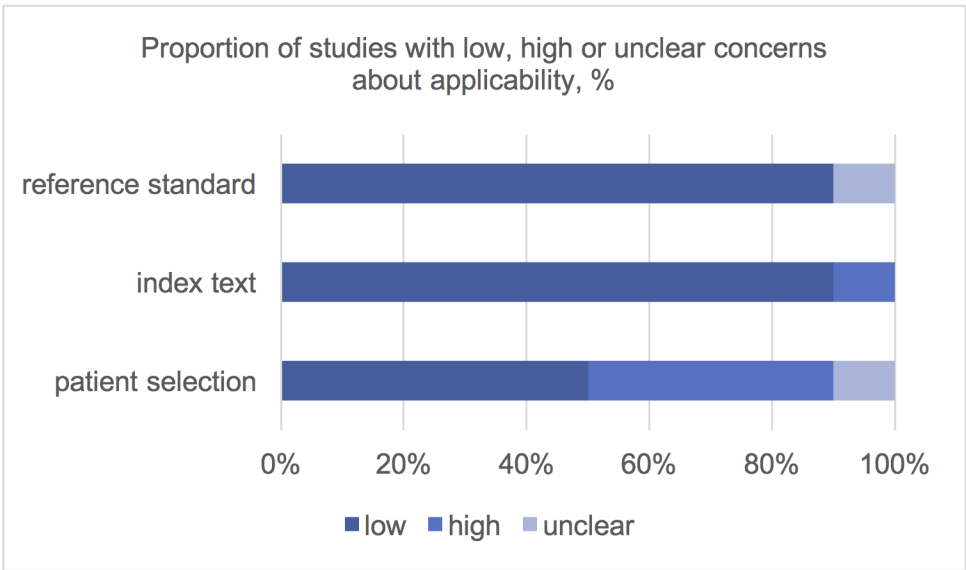

Supplement: Supplementary file 1 [file CAM4-8-480-s001.pdf]

Figure S2: Publication bias of included studies

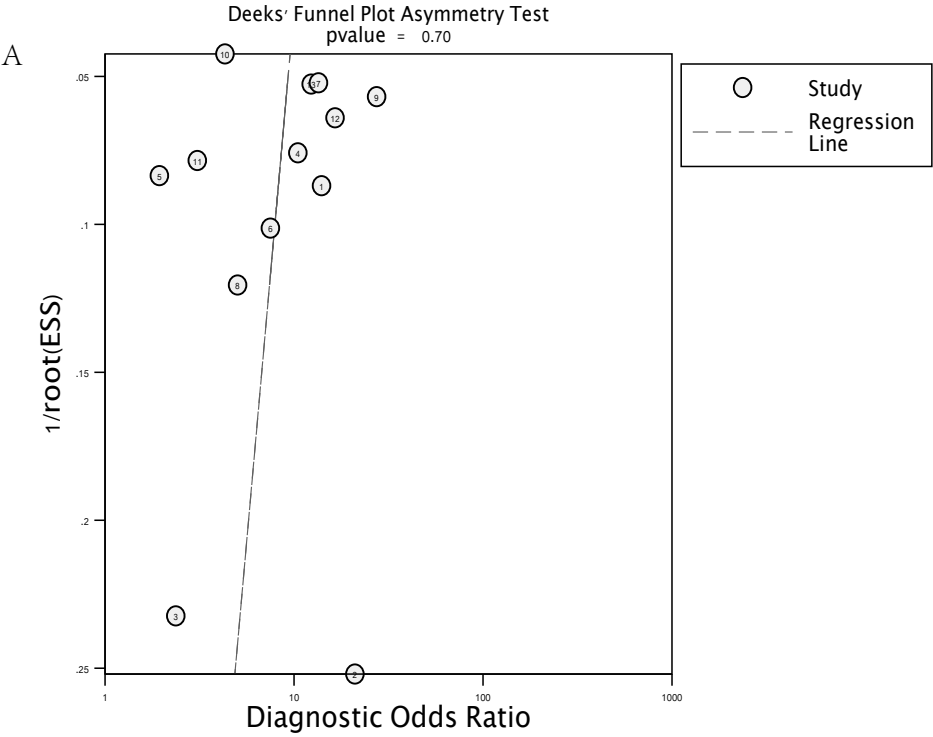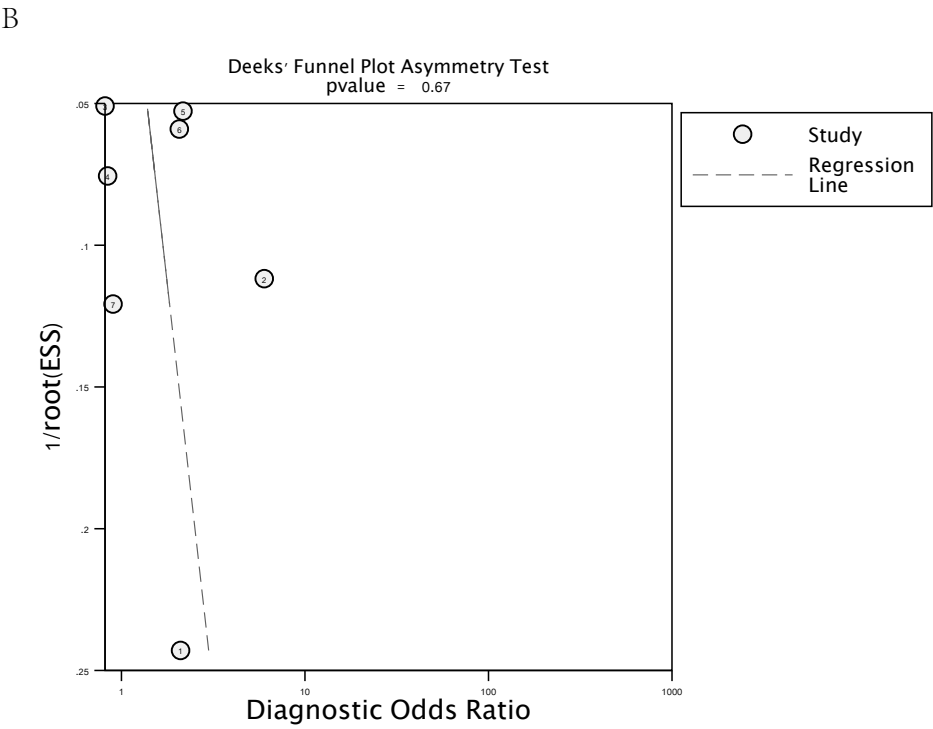

Supplement: Supplementary file 2 [file CAM4-8-480-s002.pdf]

# Figure S3: Pooled DLR of fecal *Fusobacterium nucleatum* for colorectal cancer

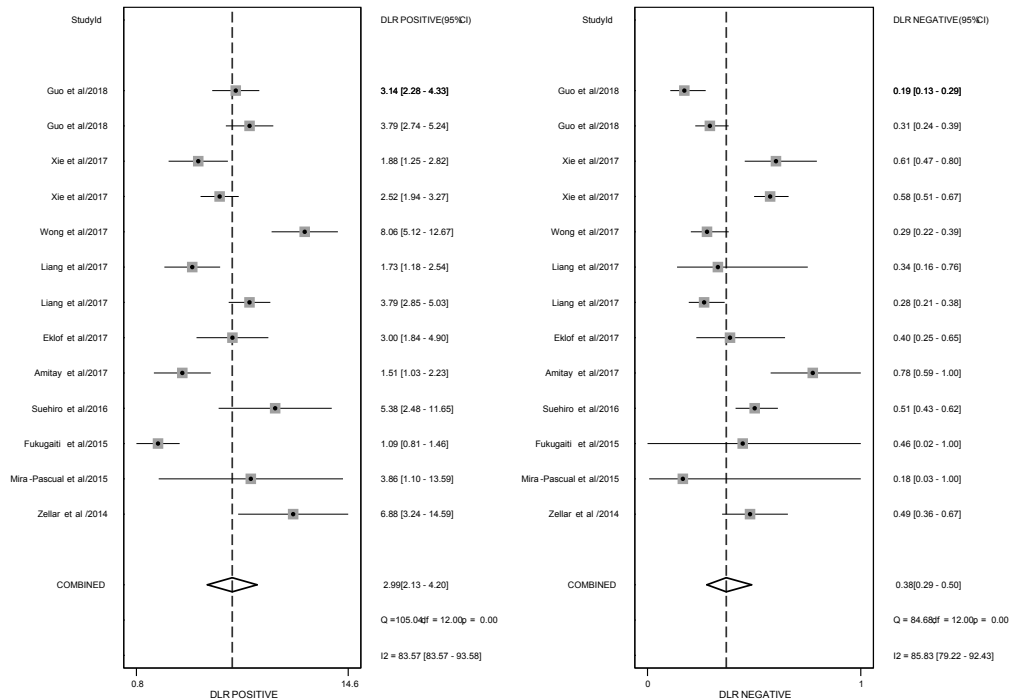

Supplement: Supplementary file 3 [file CAM4-8-480-s003.pdf]

# Figure S4: Pooled DLR of fecal *Fusobacterium nucleatum* for colorectal adenoma

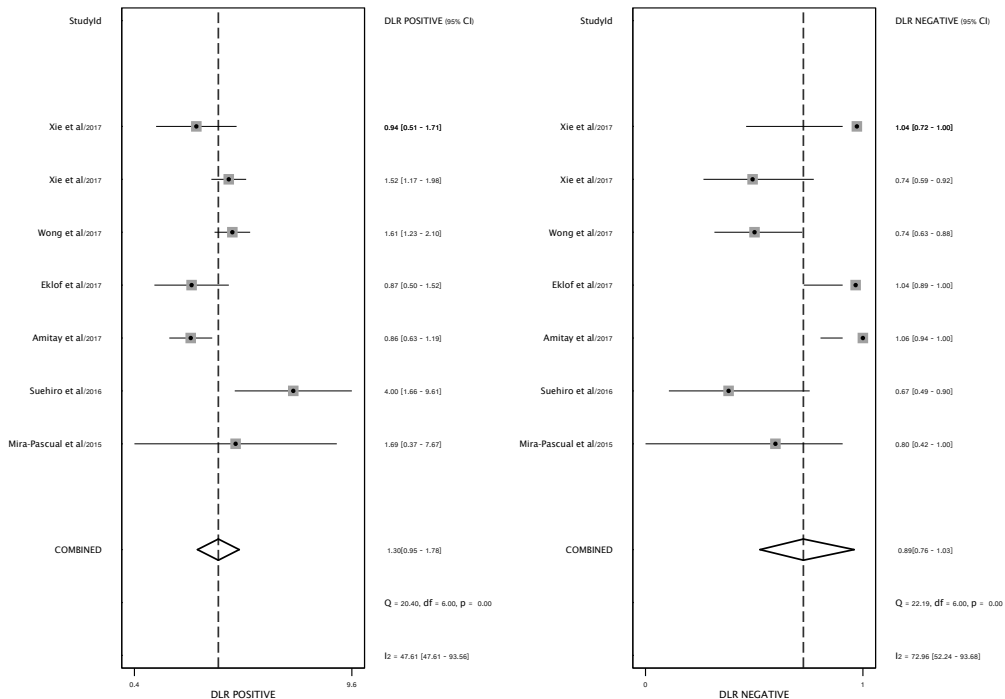

Supplement: Supplementary file 4 [file CAM4-8-480-s004.pdf]
