# Supplementary material for: Fecal Fusobacterium nucleatum for the diagnosis of colorectal tumor: A systematic review and meta‐analysis
Source: Cancer Med. 2019 Jan 12;8(2):480–91. doi: 10.1002/cam4.1850 (PMC6382715; doi:10.1002/cam4.1850)
Supplement: Supplementary file 5 [file CAM4-8-480-s005.docx]

Table S1: QADAS-2 results for included studies

| **Author, Year** | **Risk of Bias** | | | | **Applicability Concerns** | | |
| --- | --- | --- | --- | --- | --- | --- | --- |
|  | Patient Selection | Index Text | Reference Standard | Flow and Timing | Patient Selection | Index Text | Reference Standard |
| Zellar et al^24^,2014 | High | Low | Low | Low | High | High | Low |
| Mira-Pascual et al^32^,2015 | Low | High | Low | Low | Low | Low | Low |
| Fukugaiti et al^19^,2015 | High | High | Low | Low | Low | Low | Low |
| Suehiro et al^31^,2016 | High | High | Unclear | Unclear | Unclear | Low | Low |
| Wong et al^22^,2017 | High | High | Low | Low | High | Low | Low |
| Eklof et al^20^,2017 | Low | High | Low | Low | Low | Low | Low |
| Amitay et al^23^,2017 | High | High | Low | Low | Low | Low | Low |
| Liang et al^21^,2017 | High | High | Low | Unclear | High | Low | Low |
| Xie et al^25^,2017 | High | High | Low | Unclear | High | Low | Low |
| Guo et al^33^,2018 | High | High | Low | Low | Low | Low | Low |
| Guo et al^33^,2018 | High | High | Low | Low | Low | Low | Low |
