# Supplementary material for: Fecal Fusobacterium nucleatum for the diagnosis of colorectal tumor: A systematic review and meta‐analysis
Source: Cancer Med. 2019 Jan 12;8(2):480–91. doi: 10.1002/cam4.1850 (PMC6382715; doi:10.1002/cam4.1850)
Supplement: Supplementary file 6 [file CAM4-8-480-s006.docx]

Table S2: Results of meta-regression for CRC

| **Parameter** | **Catergory** | **Cohorts, n** | **Sensitivity** | **P1** | **Specificity** | **P2** | **P value** |
| --- | --- | --- | --- | --- | --- | --- | --- |
| **Early stage^a^** | Yes | 5 | 0.65 [0.54 - 0.76] | 0.52 | 0.79 [0.70 - 0.89] | 0.19 | 0.00 |
|  | No | 4 | 0.61 [0.49 - 0.73] |  | 0.76 [0.65 - 0.87] |  |  |
| **CRC^b^** | Yes | 5 | 0.67 [0.53 - 0.80] | 0.09 | 0.79 [0.67 - 0.92] | 0.47 | 0.73 |
|  | No | 8 | 0.74 [0.62 - 0.85] |  | 0.74 [0.62 - 0.87] |  |  |
| **Male^c^** | Yes | 4 | 0.77 [0.61 - 0.93] | 0.72 | 0.69 [0.49 - 0.90] | 0.20 | 0.00 |
|  | No | 8 | 0.70 [0.59 - 0.81] |  | 0.77 [0.66 - 0.87] |  |  |
| **Age^d^** | Yes | 7 | 0.66 [0.55 - 0.77] | 0.01 | 0.82 [0.73 - 0.92] | 0.93 | 0.00 |
|  | No | 5 | 0.80 [0.70 - 0.91] |  | 0.68 [0.51 - 0.85] |  |  |

a：The percent of early-stage patients in all CRC patients is over 50%. b: The percent of CRC patients in all participants is over 50%. c: The percent of males in all participants is over 60%. d: The average age of all participants is over 60 years old. P1: The univariable regression results of sensitivity. P2: The univariable regression results of specificity. P value: The joint model regression results of the overall heterogeneity.
