# Supplementary material for: Fecal Fusobacterium nucleatum for the diagnosis of colorectal tumor: A systematic review and meta‐analysis
Source: Cancer Med. 2019 Jan 12;8(2):480–91. doi: 10.1002/cam4.1850 (PMC6382715; doi:10.1002/cam4.1850)
Supplement: Supplementary file 7 [file CAM4-8-480-s007.docx]

**Supplementary Method. Search Strategies**

**PUBMED**

#1 (((((fecal[MeSH Terms]) OR fecal[Text Word]) OR faecal[MeSH Terms]) OR faecal[Text Word]) OR feces[MeSH Terms]) OR fece*[Text Word]

#2 ((fusobacterium nucleatum[MeSH Terms]) OR fusobacteri*[Text Word]) OR F.nucleatum[Text Word]

#3 ((((fusobacterium nucleatum[MeSH Terms]) OR fusobacteri*[Text Word]) OR F.nucleatum[Text Word])) AND ((((((fecal[MeSH Terms]) OR fecal[Text Word]) OR faecal[MeSH Terms]) OR faecal[Text Word]) OR feces[MeSH Terms]) OR fece*[Text Word])

#4 (((((colorectal neoplasms[MeSH Terms]) OR colonic neoplasms[MeSH Terms]) OR rectum neoplasms[MeSH Terms]) OR sigmoid neoplasms[MeSH Terms]) OR colonic polyps[MeSH Terms]) OR intestinal polyps[MeSH Terms]

#5 (((((colorectal[Text Word]) OR colon*[Text Word]) OR rect*[Text Word]) OR intestin*[Text Word]) OR sigmoid[Text Word]) OR anal[Text Word]

#6 ((((((cancer*[Text Word]) OR tumor*[Text Word]) OR tumour*[Text Word]) OR adenoma*[Text Word]) OR polyp*[Text Word]) OR carcinoma*[Text Word]) OR neoplas*[Text Word]

#7 (((((((colorectal[Text Word]) OR colon*[Text Word]) OR rect*[Text Word]) OR intestin*[Text Word]) OR sigmoid[Text Word]) OR anal[Text Word])) AND (((((((cancer*[Text Word]) OR tumor*[Text Word]) OR tumour*[Text Word]) OR adenoma*[Text Word]) OR polyp*[Text Word]) OR carcinoma*[Text Word]) OR neoplas*[Text Word])

#8 (((((((colorectal neoplasms[MeSH Terms]) OR colonic neoplasms[MeSH Terms]) OR rectum neoplasms[MeSH Terms]) OR sigmoid neoplasms[MeSH Terms]) OR colonic polyps[MeSH Terms]) OR intestinal polyps[MeSH Terms])) OR ((((((((colorectal[Text Word]) OR colon*[Text Word]) OR rect*[Text Word]) OR intestin*[Text Word]) OR sigmoid[Text Word]) OR anal[Text Word])) AND (((((((cancer*[Text Word]) OR tumor*[Text Word]) OR tumour*[Text Word]) OR adenoma*[Text Word]) OR polyp*[Text Word]) OR carcinoma*[Text Word]) OR neoplas*[Text Word]))

#9 (((((((((colorectal neoplasms[MeSH Terms]) OR colonic neoplasms[MeSH Terms]) OR rectum neoplasms[MeSH Terms]) OR sigmoid neoplasms[MeSH Terms]) OR colonic polyps[MeSH Terms]) OR intestinal polyps[MeSH Terms])) OR ((((((((colorectal[Text Word]) OR colon*[Text Word]) OR rect*[Text Word]) OR intestin*[Text Word]) OR sigmoid[Text Word]) OR anal[Text Word])) AND (((((((cancer*[Text Word]) OR tumor*[Text Word]) OR tumour*[Text Word]) OR adenoma*[Text Word]) OR polyp*[Text Word]) OR carcinoma*[Text Word]) OR neoplas*[Text Word])))) AND (((((fusobacterium nucleatum[MeSH Terms]) OR fusobacteri*[Text Word]) OR F.nucleatum[Text Word])) AND ((((((fecal[MeSH Terms]) OR fecal[Text Word]) OR faecal[MeSH Terms]) OR faecal[Text Word]) OR feces[MeSH Terms]) OR fece*[Text Word]))

#10 ((((((((((((((((((((sensitivity and specificity[MeSH Terms])) OR diagnoses[MeSH Terms]) OR likelihood functions[MeSH Terms]) OR area under curve[MeSH Terms]) OR predictive value of tests[MeSH Terms]) OR false negative reactions[MeSH Terms]) OR false positive reactions[MeSH Terms]) OR diagnostic errors[MeSH Terms]) OR diagnos*[Text Word]) OR screen*[Text Word]) OR detect*[Text Word]) OR recogni*[Text Word]) OR false positiv*[Text Word]) OR false negativ*[Text Word]) OR true negativ*[Text Word]) OR true negativ*[Text Word]) OR likelihood ratio*[Text Word]) OR accurac*[Text Word]) OR sensitiv*[Text Word]) OR specific*[Text Word]

#11 (((((((((((colorectal neoplasms[MeSH Terms]) OR colonic neoplasms[MeSH Terms]) OR rectum neoplasms[MeSH Terms]) OR sigmoid neoplasms[MeSH Terms]) OR colonic polyps[MeSH Terms]) OR intestinal polyps[MeSH Terms])) OR ((((((((colorectal[Text Word]) OR colon*[Text Word]) OR rect*[Text Word]) OR intestin*[Text Word]) OR sigmoid[Text Word]) OR anal[Text Word])) AND (((((((cancer*[Text Word]) OR tumor*[Text Word]) OR tumour*[Text Word]) OR adenoma*[Text Word]) OR polyp*[Text Word]) OR carcinoma*[Text Word]) OR neoplas*[Text Word])))) AND (((((fusobacterium nucleatum[MeSH Terms]) OR fusobacteri*[Text Word]) OR F.nucleatum[Text Word])) AND ((((((fecal[MeSH Terms]) OR fecal[Text Word]) OR faecal[MeSH Terms]) OR faecal[Text Word]) OR feces[MeSH Terms]) OR fece*[Text Word])))) AND (((((((((((((((((((((sensitivity and specificity[MeSH Terms])) OR diagnoses[MeSH Terms]) OR likelihood functions[MeSH Terms]) OR area under curve[MeSH Terms]) OR predictive value of tests[MeSH Terms]) OR false negative reactions[MeSH Terms]) OR false positive reactions[MeSH Terms]) OR diagnostic errors[MeSH Terms]) OR diagnos*[Text Word]) OR screen*[Text Word]) OR detect*[Text Word]) OR recogni*[Text Word]) OR false positiv*[Text Word]) OR false negativ*[Text Word]) OR true negativ*[Text Word]) OR true negativ*[Text Word]) OR likelihood ratio*[Text Word]) OR accurac*[Text Word]) OR sensitiv*[Text Word]) OR specific*[Text Word])

**Cochrane library**

#1 "fecal" or "faecal" or fece or stool (Word variations have been searched)

#2 MeSH descriptor: [Colorectal Neoplasms] explode all trees

#3 MeSH descriptor: [Intestinal Polyps] explode all trees

#4 MeSH descriptor: [Colonic Polyps] explode all trees

#5 MeSH descriptor: [Colonic Neoplasms] explode all trees

#6 MeSH descriptor: [Sigmoid Neoplasms] explode all trees

#7 colon* or colorectal or sigmoid or anal or rect* (Word variations have been searched)

#8 adenoma* or polyp or carcinoma* or neoplas* or tumor* (Word variations have been searched)

#9 tumor* or cancer* (Word variations have been searched)

#10 #8 or #9

#11 MeSH descriptor: [Rectal Neoplasms] explode all trees

#12 #2 or #3 or #4 or #5 or #6 or #11

#13 #7 and #10

#14 #12 or #13

#15 MeSH descriptor: [Fusobacterium nucleatum] explode all trees

#16 "Fusobacterium nucleatum" or fusobacteri* or F.nucleatum (Word variations have been searched)

#17 MeSH descriptor: [Sensitivity and Specificity] explode all trees

#18 MeSH descriptor: [Likelihood Functions] explode all trees

#19 MeSH descriptor: [Area Under Curve] explode all trees

#20 MeSH descriptor: [Predictive Value of Tests] explode all trees

#21 MeSH descriptor: [False Negative Reactions] explode all trees

#22 MeSH descriptor: [False Positive Reactions] explode all trees

#23 MeSH descriptor: [Diagnosis] explode all trees

#24 MeSH descriptor: [Diagnostic Errors] explode all trees

#25 sensitiv* or specificit* or accurac* or likelihood ratio (Word variations have been searched)

#26 false positiv* or false negativ* or true positiv* or true negativ* (Word variations have been searched)

#27 diagnos* or screen* or detect* or recogni* (Word variations have been searched)

#28 #17 or #18 or #19 or #20 or #21 or #22 or #23 or #24 or #25 or #26 or #27

#29 #15 or #16

#30 #1 and #14 and #28 and #29

**Embase**

#1 fecal OR faecal OR feces

#2 'fusobacterium nucleatum' OR fusobacteri* OR f.nucleatum

#3 'colorectal tumor' OR 'colon tumor' OR 'rectum tumor' OR 'colorectal carcinoma' OR 'colon carcinoma' OR 'rectum carcinoma' OR 'colorectal cancer' OR 'colon cancer' OR 'rectum cancer' OR 'intestine polyp' OR 'colon polyp' OR 'colon neoplasms'

#4 colorect* OR colon* OR rect* OR anal* OR intestin* OR adenomat* OR sigmoid

#5 adenoma* OR polyp* OR carcinoma* OR tumo* OR neoplas* OR adenocarcinoma*

#6 #4 AND #5

#7 #3 OR #6

#8 #1 AND #2

#9 #7 AND #8

#10 'sensitivity and specificity' OR diagnosis OR 'diagnostic error' OR 'diagnostic value' OR 'diagnostic accuracy' OR 'diagnostic test accuracy study' OR 'predictive value' OR 'laboratory diagnosis' OR 'area under the curve' OR 'reference value' OR detect* OR screen* OR recogni* OR sensitiv* OR specificit* OR accurac* OR 'likelihood ratio' OR (false AND negativ*) OR (false AND positiv*) OR 'receiver operating characteristic' OR 'diagnostic odds ratio'

#11 #9 AND #10

**Web of science**

#1 (fecal) OR (faecal) OR (fece*)

#2 (fusobacterium nucleatum) OR (fusobacteri*) OR (F.nucleatum)

#3 #2 AND #1

#4 (colorectal) OR (colon*) OR (rect*) OR (sigmoid) OR (anal) OR (colorectal) OR (intestin*)

#5 (cancer*) OR (tumor*) OR (tumour*) OR (adenoma*) OR (neoplas*) OR (carcinoma*) OR (polyp*)

#6 #5 AND #4

#7 (diagnos*) OR (sensitiv*) OR (specific*) OR (screen*) OR (detect*)

#8 #7 AND #6 AND #3
